# Quantitative real-time *in vitro* transcription assay (QRIVTA) for transcriptional regulation studies

**DOI:** 10.1093/procel/pwae054

**Published:** 2024-10-10

**Authors:** Fan Liu, Jing Xu, Xinli Hu, Bo Duan, Bin Xia

**Affiliations:** School of Life Sciences, Peking University, Beijing 100871, China; Beijing Nuclear Magnetic Resonance Center, Peking University, Beijing 100871, China; School of Life Sciences, Peking University, Beijing 100871, China; Beijing Nuclear Magnetic Resonance Center, Peking University, Beijing 100871, China; Institute of Molecular Medicine, College of Future Technology, Peking University, Beijing 100871, China; Suzhou Key Laboratory of Pathogen Bioscience and Anti‑infective Medicine, MOE Key Laboratory of Geriatric Diseases and Immunology, Institute of Molecular Enzymology, School of Life Sciences, Soochow University, Suzhou 215123, China; School of Life Sciences, Peking University, Beijing 100871, China; Beijing Nuclear Magnetic Resonance Center, Peking University, Beijing 100871, China; College of Chemistry and Molecular Engineering, Peking University, Beijing 100871, China

**Dear Editor,**

The *in vitro* transcription (IVT) assay is a powerful tool frequently used in dissecting the molecular mechanism of transcriptional regulation and also plays an important role in the field of drug discovery and RNA-based therapeutics (Yang and Ma, 2016). Traditionally, the IVT assay adopted the incorporation of radioactive nucleotides for RNA detection, which requires time-consuming RNA isolation and PAGE analysis steps, as well as extra safety measures (Höfer et al., 2013; Yang and Ma, 2016). In addition, it could only be used to evaluate the transcription regulation qualitatively or semi-quantitatively. In recent years, several strategies have been proposed to develop real-time IVT (RT-IVT) assays based on fluorescence detection with fluorophore-labeled antisense probes such as the molecular beacon (MB) (Marras et al., 2004) or the fluorescent light-up RNA aptamer (Höfer et al., 2013; Huang et al., 2022; Jensen et al., 2023; Qin et al., 2022). The MB-based detection strategy is to use a single-stranded oligonucleotide with a stem-loop structure, which contains a fluorescent group in the 5ʹ end and a quencher group in the 3ʹ end. Upon hybridizing with the RNA target, the stem-loop structure of MB unfolds, and thus, the fluorophore is separated from the quencher, resulting in the activation of the fluorescence (Fig. S1). The RNA aptamer-based detection strategy is to introduce an RNA aptamer sequence into the target RNA, where the aptamer adopts a defined structure and can specifically bind to and activate the fluorescence of a fluorophore, such as the small molecule mimic of green fluorescent protein (DFHBI) (Fig. S1). These fluorescence detection strategies can be used for quantitative analysis and have a promising prospect in high-throughput and automatic detection. However, despite their great potential, the fluorescence-based RT-IVT assays are still not well established and rarely used in transcription-related studies nowadays. A major reason is the lack of a standard experimental workflow and data analysis method to ensure good data repeatability, reproducibility, and comparability.

In this study, we introduce a quantitative real-time *in vitro* transcription assay (QRIVTA) for transcription and transcriptional regulation studies, which can be easily carried out with a common real-time PCR thermocycler, along with guidelines for carrying out IVT experiments. It is observed that the fluorescence production rate is attenuated with time when using supercoiled plasmids as transcription templates, even in the presence of excessive rNTPs and MB. We show that the attenuation should reflect the topological characteristics of the supercoiled plasmid template. A new equation for quantitative analysis is proposed to precisely describe the entire time-dependent attenuating fluorescence data from QRIVTA using supercoiled plasmid templates. The transcription repression of the global transcription regulator H-NS on the promoter region of pathogenic *E*. *coli LEE5* operon is used as a model system. Both the MB-based and RNA aptamer-based detection strategies are leveraged to optimize and develop the assay. We have systematically evaluated and optimized different experimental aspects of the fluorescence-based RT-IVT assay regarding plasmid template design, fluorescence detection strategy, sensitivity and specificity tests, experimental operation, data analysis, etc., which enable us to propose a standardized workflow and detailed practical guidelines for carrying out QRIVTA in transcriptional regulation studies.

We cloned the −304 to +171 sequences of the *LEE5* promoter (defined as *LEE5p* gene) (Shin, 2017), followed by a molecular beacon (MB1) complementary sequence (Seq_MB1_) (Dong et al., 2018) and a 47-bp *E*. *coli rrnB T*_1_ terminator sequence (*rrnBT*_1__47), into a modified pUC19 plasmid (pUC19s) (Fig. S2A). Two plasmids were constructed, with the *LEE5p* oriented in a divergent (*trans*-pLEE5p) or tandem (*cis*-pLEE5p) direction relative to the *AmpR* gene (Fig. S2A).

Due to the weak fluorescence observed with MB1 during the RT-IVT assays, we designed a new molecular beacon (MB2) with its loop sequence complementary to the +129 to +146 region (Seq_MB2_) of the *LEE5p* (Fig. S3), which exhibited much stronger fluorescence. For RT-IVT assays using the supercoiled *cis*-pLEE5p template, the fluorescence production rate is attenuated with time, regardless of the template concentration, and no increase in fluorescence without the DNA template, as expected (Fig. S2B). Since further increasing the template concentration didn't substantially enhance fluorescence intensity, 10 nmol/L was adopted for subsequent investigations. For the *trans*-pLEE5p template, the fluorescence production rate is also attenuated significantly. However, it slows down very quickly and plateaus after 30 min, while the *cis*-pLEE5p template shows slower attenuation and robust fluorescence increase over 120 min (Fig. S2C). We next examined the specificity of MB2 using modified *cis*-pLEE5p templates with Seq_MB2_ mutated or deleted, detecting −8% nonspecific fluorescence (Fig. S2C), which may be resulted from the high GC content and the presence of 5-bp or more consecutive G/C stretches in the loop region of MB2 (Fig. S3).

We also carried out the RT-IVT assays using aptamer-based detection. Seq_MB1_ in the *cis*-pLEE5p was substituted with either the Spinach (Jensen et al., 2023) or iSpinach (Qin et al., 2022) sequence ( Fig. S4A), and the fluorescence of aptamer-bound DFHBI was monitored. iSpinach exhibits higher fluorescence than Spinach at low DFHBI concentrations (Fig. S4B and S4C), and both aptamers display much weaker fluorescence compared with that of MB2. Notably, the iSpinach-based detection demonstrates high specificity without nonspecific fluorescence when using a *cis*-pLEE5p template lacking the iSpinach sequence (Fig. S4D). Then, the iSpinach-based detection was applied to evaluate the transcription termination efficiency of *LEE5p* by placing the iSpinach sequence downstream of the terminator (Fig. S5A). The *rrnBT*_1__47 terminator shows a termination efficiency of 93% (Figs. 1A and S5B). Five previously reported terminators with high efficiency were tested (Cambray et al., 2013; Chen et al., 2013), and only the M13D double terminator (M13 central and *rrnD T*_1_) exhibits particularly high efficiency (~99%) (Figs. 1A and S5B). The *rrnBT*_1__91 terminator barely improves the efficiency to 95%, whereas all the others fall below 90%. As the tandem concatenation of terminators could enhance the termination efficiency (Mairhofer et al., 2015), we designed the RBT double terminator (*rrnBT*_1__47 and BBaU10), which also exhibits a superior termination efficiency (~99%) (Figs. 1A and S5B). The transcription of *AmpR* was also examined using the iSpinach-based detection ( Fig. S6A), which is less than one-tenth that of *LEE5p* (Fig. S6B). To avoid transcriptional read-through of *AmpR*, an *rrnBT*_1__47 terminator sequence was inserted between *AmpR* and iSpinach (Fig. S6A), which effectively terminates its transcription (Fig. S6B).

**Figure 1.**
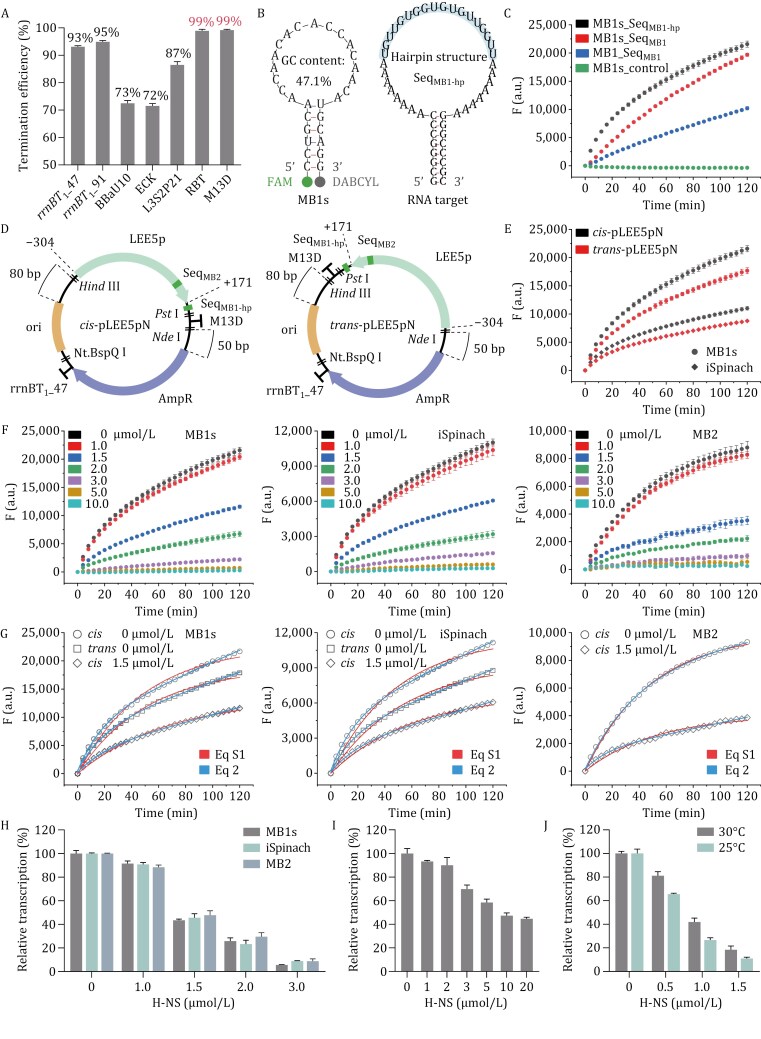
Development and optimization of QRIVTA. (A) Termination efficiency of transcription terminators. The 91-bp “*rrnB T*_1_” (*rrnBT*_1__91), “M13 central + *rrnD T*_1_” (M13D) and BBa_B1006 U10 (BBaU10) (Cambray et al., 2013), as well as ECK120029600 (ECK) and L3S2P21 (Chen et al., 2013) are previously reported terminators with high efficiency. The 47-bp “*rrnB T*_1_” (*rrnBT*_1__47) and “*rrnBT*_1__47 + BBaU10” (RBT) are newly designed terminators in this study. The termination efficiency is calculated by subtracting 100% from the percentage of transcription read-through, which is defined as the ratio between fluorescence intensities detected at 120 min from plasmid templates with the iSpinach sequence positioned downstream and upstream of the terminator. The termination efficiency and error bar represent the mean and standard deviation (*n* = 3), respectively. (B) Secondary structures of the MB1s MB and Seq_MB1-hp_ RNA, with the MB1s complementary sequence Seq_MB1_ highlighted. (C) Time courses of the fluorescence production from RT-IVT assays with 10 nmol/L supercoiled plasmid template at 37°C, using MB1- or MB1s-based detection. The design of the plasmid with Seq_MB1_ is shown in Fig. S6A, and Seq_MB1_ is replaced by Seq_MB1-hp_ in the plasmid with Seq_MB1-hp_. The control plasmid template is devoid of either Seq_MB1-hp_ or Seq_MB1_. Each fluorescence data point is the average of three replicates, with the error bar indicating the standard deviation. (D) Plasmid design of *cis*-pLEE5pN and *trans*-pLEE5pN. (E) Time courses of the fluorescence production from RT-IVT assays with 10 nmol/L supercoiled *cis*-pLEE5pN (black) and *trans*-pLEE5pN (red) templates at 37°C, using MB1s- (circle) or iSpinach- (diamond) based detection. For iSpinach/DFHBI fluorescence detection, Seq_MB1-hp_ in the plasmids is substituted with the iSpinach sequence. Each fluorescence data point is the average of three replicates, with the error bar indicating the standard deviation. (F) Time courses of the fluorescence production from RT-IVT assays with 10 nmol/L supercoiled plasmid template at 37°C, in the absence or presence of H-NS at indicated concentrations, using MB1s- (left), iSpinach- (middle), or MB2- (right) based detection. The plasmid *cis*-pLEE5pN is used for MB1s-based detection. For iSpinach-based detection, Seq_MB1-hp_ in *cis*-pLEE5pN is substituted with the iSpinach sequence. For MB2-based detection, the plasmid design is depicted in Fig. S12A. Each fluorescence data point is the average of three replicates, with the error bar indicating the standard deviation. (G) Representative fitting curves using Equation 2 (blue line) and Equation S1 (red line) for fluorescence data from RT-IVT assays mentioned in (f). Open circle and diamond indicate data from RT-IVT assays using *cis* templates with 0 and 1.5 μmol/L H-NS, respectively. An open square indicates data from RT-IVT assays using *trans* templates. (H) Comparison of the transcription repression ability of H-NS on supercoiled plasmid template from RT-IVT assays using MB1s-, iSpinach-, or MB2-based detection strategies. (I) The transcription repression ability of H-NS on linearized *cis*-pLEE5pN template. (J) Effect of temperature on the transcription repression ability of H-NS on supercoiled *cis*-pLEE5pN template. In (H–J), the relative transcription in the presence of H-NS at indicated concentrations is defined as the percentage of the initial fluorescence production rate relative to that without H-NS. Each percentage of relative transcription and error bar represent the mean and standard deviation (*n* = 3), respectively.

Due to the nonspecific issue with MB2, we refocused on MB1 after the optimization of terminators. The loop region of MB1, with a lower GC content (47.1%) and no long consecutive G/C stretch (Fig. S3), is less likely to cause nonspecific interactions. To optimize the fluorescence intensity of MB1, we modified its design and target RNA sequence by first removing one GC pair from the stem region of MB1 (MB1s) to facilitate its unwinding (Fig. 1B). Then, its target sequence Seq_MB1_ was positioned in a loop region of a designed hairpin structure (Seq_MB1-hp_) in the transcription template (Fig. 1B), to improve the accessibility of Seq_MB1_ on the target RNA. As expected, the modifications result in significantly enhanced detection sensitivity and high specificity, with no nonspecific fluorescence from MB1s when using a template lacking Seq_MB1-hp_ (Fig. 1C).

With all the above optimizations, new plasmids *cis*-pLEE5p (*cis*-pLEE5pN) and *trans*-pLEE5p (*trans*-pLEE5pN) were constructed, with *LEE5p* followed by Seq_MB1-hp_ and the M13D terminator, positioned in a divergent or tandem orientation relative to the *AmpR* (Fig. 1D), respectively. Transcription of *LEE5p* in these plasmids was measured using either the MB1s- or iSpinach-based detection. It was found that fluorescence production rates are all attenuated over time with both detection strategies. Notably, different from the *trans*-pLEE5p, the fluorescence of the new *trans*-pLEE5pN template no longer plateaus quickly and maintains a continuous increase, although it is still weaker than that of *cis*-pLEE5pN (Fig. 1E). These results indicate that the transcription of *AmpR* in the old *trans*-pLEE5p template, which is not efficiently terminated, significantly impacts the *LEE5p* transcription.

In our RT-IVT assays, RNA polymerase (RNAP) substrates rNTPs (0.5 mmol/L) and MB (500 nmol/L) are used in vast excess (Fig. S7); their consumption should not be the reason for the attenuation of fluorescence production rate. Interestingly, both linearized and nicked *cis*-pLEE5pN templates resulted in a linear increase in fluorescence intensity (Figs. S8 and S9), indicating that the attenuation should also not be caused by the RNAP stability. Therefore, the attenuation of the fluorescence production rate should reflect some properties of the supercoiled plasmid template during transcription. It is well established that the transcription would generate positive supercoils in front of the RNAP elongation complex and negative supercoils behind on the DNA template, which was termed the "twin supercoiled domain model" (Chong et al., 2014). The positive supercoils accumulated in front of the RNAP elongation complex could slow down the transcription elongation, thus attenuating the RNA production rate over time. In addition, transcriptional pausing can cause RNAP stalling or being trapped on the supercoiled DNA template, which could also decelerate the transcription rate (Huang et al., 2022).

To evaluate the transcription efficiency in RT-IVT assays using supercoiled plasmids as transcription templates, it is more general to determine the initial fluorescence production rate (*v*_0_) from the fluorescence curve, which is the maximal fluorescence production rate during the time course of the IVT assay, reflecting the maximal steady-state transcription rate. We first tested fitting the fluorescence curves using an exponential decay (increase form) function (Equation S1 in materials and methods) (Huang et al., 2022), but it did not produce very good curve fitting results for the data from the MB1s- or iSpinach-based detection (Fig. 1G). We then analyzed the first derivatives of the fluorescence data (Fig. S10), which reflect the fluorescence production rate at each time point. It appears that the production rate does not go to zero at the end of detection, even though it is attenuated with time. Therefore, the first derivative data can be well-fitted to an equation (Fig. 2A, Equation 1) with an exponentially decaying rate *v*_d_(*t*) and a constant rate *v*_c_ (Fig. S10). Using the indefinite integral, we can derive the function of fluorescence intensity with respect to time (Fig. 2A; Equation 2). *v*_0_ is thus the sum of *v*_d0_ and *v*_c_ (*v*_0_ = *v*_d0_ + *v*_c_) (Fig. 2A).

**Figure 2.**
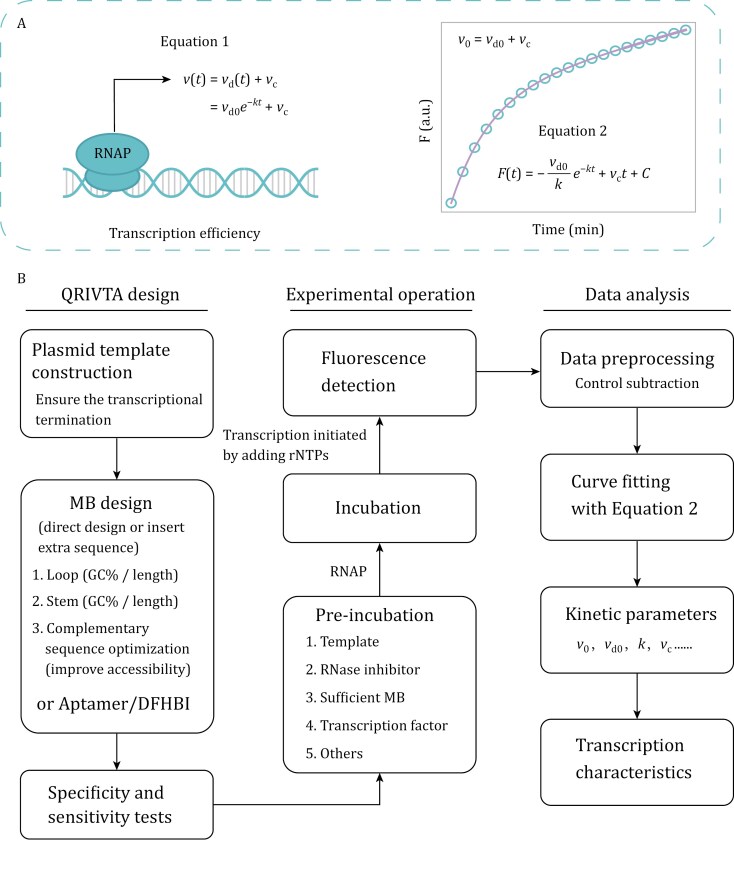
Illustration of the concept and workflow of QRIVTA. (A) Diagram of the fluorescence data analysis in QRIVTA. Equation 1 directly represents the relationship between the fluorescence production rate and time. This rate is composed of an exponentially decaying rate *v*_d_(*t*) and a constant rate *v*_c_. *v*_d0_ is the initial rate for *v*_d_(*t*), and *k* is the attenuation rate constant of *v*_d_(*t*). Equation 2 is the function of fluorescence intensity with respect to time, derived from Equation 1 through the indefinite integral, where C is the integration constant. (B) Diagram of the QRIVTA workflow.

The transcription repression ability of H-NS on *LEE5p* was evaluated using both the MB1s- and iSpinach-based detection with the supercoiled *cis*-pLEE5pN template. A concentration-dependent decrease in fluorescence production rate is observed with the increase of H-NS concentration (Fig. 1F). Remarkably, all the fluorescence data of the *LEE5p* transcription obtained with or without H-NS, as well as the fluorescence data of the *AmpR* transcription, can be well fitted with Equation 2 (Figs. 1G, S6C and S11), demonstrating the robustness of this new equation.

Using Equation 2, we determined *v*_0_ from both the MB1s- and iSpinach-based detection for different H-NS concentrations (Table S4). *v*_0_ is used to reflect the transcription efficiency, and thus, the percentage of reduction in *v*_0_ reflects the transcription repression ability of H-NS. The results show that the transcription repression ability of H-NS displays a steep increase rather than a linear increase above a certain concentration. For the MB1s-based detection, a slight transcription repression (9%) is observed with 1 μmol/L H-NS (Fig. 1H), which is markedly enhanced upon increasing the H-NS concentration from 1 to 3 μmol/L. Specifically, *v*_0_ is repressed by 57% at 1.5 μmol/L, 74% at 2 μmol/L, and 94% at 3 μmol/L H-NS, respectively (Fig. 1H). Further increasing the concentration to 5 and 10 μmol/L results in nearly complete transcription repression. This is consistent with that H-NS oligomerizes in a concentration-dependent manner, which only forms higher-order oligomers at higher concentrations, and the DNA binding affinity of H-NS is intrinsically associated with its oligomerization level (Lukose et al., 2024). At the same concentration, the transcription repression ability of H-NS determined from the iSpinach-based detection is about the same as that of MB1s (Fig. 1H).

To investigate the potential impact of extra sequence downstream of the *LEE5p* on transcription repression of H-NS, we removed the Seq_MB1-hp_ from the *cis*-pLEE5pN template and used MB2-based detection to measure the *LEE5p* transcription (Figs. 1F and S12). Nonspecific fluorescence of MB2 was measured using a Seq_MB2_-mutated plasmid template under identical conditions, and specific fluorescence was obtained by subtracting nonspecific fluorescence from the raw fluorescence (Fig. S12B). Notably, all the fluorescence data from the MB2-based detection can also be well-fitted with Equation 2 (Fig. 1G). The transcription repression ability of H-NS determined from the MB2-based detection is also essentially the same as those determined from the MB1s- and iSpinach-based detection (Fig. 1H). Collectively, we developed an efficient and robust QRIVTA for the IVT and transcriptional regulation studies.

The repression ability of H-NS on the linearized *cis*-pLEE5pN (L1) template was also evaluated using QRIVTA (Fig. S13), with *v*_0_ obtained through linear regression. 2 μmol/L H-NS only represses *v*_0_ by 10% for the linearized plasmid template (Fig. 1I), compared to 74% for the supercoiled plasmid template (Fig. 1H), indicating that H-NS has a much stronger transcription repression ability on the supercoiled plasmid template. This is different from a previous report, which claimed that the transcription repressions of H-NS on both the linear and supercoiled DNA are the same, based on the traditional IVT assay (Shin et al., 2012). As a nucleoid-associated protein, it is reasonable that H-NS bridges or condenses supercoiled DNA more effectively, thus enhancing its repression ability.

As it is well known that temperature affects the transcription repression ability of H-NS (Lukose et al., 2024), we measured the transcription repression ability of H-NS at 30°C and 25°C (Fig. S14). Again, all the fluorescence data at lower temperatures could also be well-fitted with Equation 2. H-NS exhibits stronger repression abilities at lower temperatures with QRIVTA (Fig. 1J), which should be attributed to that H-NS forms higher-order oligomers at lower temperatures (Lukose et al., 2024). 0.5 μmol/L H-NS can repress *v*_0_ by 19% at 30°C and 34% at 25°C, compared to that 1 μmol/L H-NS only slightly represses *v*_0_ by 9% at 37°C. The transcription repression ability of 1 μmol/L H-NS is significantly enhanced to 58% at 30°C and 73% at 25°C, respectively.

It is recommended to use supercoiled plasmids as the IVT templates for the transcription regulation studies to mimic the topological state of supercoiling DNA during transcription, as short linear fragment DNA templates are less physiologically representative (Will et al., 2014). For the first time, we proposed a more precise equation (Equation 2) to accurately extract the initial rate (*v*_0_) of the fluorescence production and evaluate the transcription efficiency with supercoiled plasmid as the transcription template. Remarkably, all the fluorescence data from supercoiled plasmid templates in our study can be precisely fitted with this equation, irrespective of the detection strategy, the presence or absence of H-NS, or the experiment temperatures. More studies are required to illustrate the physical meanings of the other kinetic parameters (*k*, *v*_d0_, *v*_c_) determined from the equation.

Recently, Jensen et al. observed in their Spinach-based RT-IVT assay that the fluorescence curve shows an initial lag phase with a slow increase in fluorescence production rate, followed by a linear increase phase and then a time-dependent attenuation (Jensen et al., 2023). They used a custom MATLAB fitting program to extract the slope of the linear increase phase to determine the steady-state transcription rate. We found that the lag phase is primarily due to experimental procedure issues, which can be avoided by altering the procedure to initiate transcription with the addition of rNTPs to the premixed solution with all other components. When we initiated transcription by adding the DNA template, as Jensen et al. did, a lag phase appeared in the fluorescence curves for both MB1s- and iSpinach-based detection (Fig. S15A). We suspected that the lag phase observed is very likely due to that the template DNA plasmids, as macromolecules, take a longer time to diffuse and form the open complex for transcription initiation. On the contrary, rNTPs are small molecules with rapid diffusion rates, which could facilitate quick equilibrium in the samples. In addition, we found that the first derivatives of the fluorescence data do not reveal an obvious linear increase phase (Fig. S15B and S15C). Moreover, focusing on the presumed linear region of the fluorescence curves would omit the valuable transcriptional kinetics information of the entire fluorescence data. For comparison, the kinetic parameters of the IVT fluorescence data can be easily extracted with Equation 2 proposed in this study, which precisely describes the entire fluorescence data.

The QRIVTA enables the quantitative analysis of different intrinsic and extrinsic factors that influence transcription and transcriptional regulation, thereby enhancing our understanding of the underlying mechanisms. It is worth emphasizing that variations in the IVT experimental procedure can significantly affect the kinetics of the fluorescence production, which may result in poor data repeatability, reproducibility, and comparability. Consequently, it is crucial to standardize the fluorescence-based RT-IVT assay procedure. Detailed practical guidelines for carrying out QRIVTA, covering plasmid template design, fluorescence detection strategy, sensitivity and specificity tests, experimental operation, and data analysis, are provided in the Supplementary Material (Guidelines for QRIVTA).

In summary, a standardized and efficient workflow of QRIVTA is established, which can be easily applied in many aspects of studies related to transcriptional regulation (Fig. 2B). QRIVTA can be performed in 96- or 384-well plates using a common real-time PCR thermocycler widely available nowadays. We believe that the application of QRIVTA will not only advance the field of transcriptional regulation but also offer broad potential applications in areas such as synthetic biology and pharmaceutics.

## Supplementary data

Supplementary data is available at *Protein & Cell* online.

pwae054_suppl_Supplementary_Figures_S1-S15_Tables_S1-S4

## Footnotes

We appreciate Prof. Meiping Zhao, Prof. Peng Zou, Prof. Yiping Wang, Dr. Ziyu Yang, Dr. Ziqi Ren, and Chenyue Guo for their help in the fluorescence-based real-time IVT experiments. This work was supported by the National Natural Science Foundation of China (32371260 to B.X.) and the Ministry of Science and Technology of China (2016YFA0501200 to B.X.). All the authors declare no competing interests. All data are available in the main text or the Supplementary materials. Conceptualization: B.X. and F.L.; Methodology: F.L., X.H., B.D., and B.X.; Formal analysis: F.L. and B.X.; Investigation: F.L. and J.X.; Writing—original draft preparation: F.L.; Writing—review and editing: B.D., and B.X.; Funding acquisition: B.X.; Supervision: B.X.
