# Supplementary material for: Quantitative real-time in vitro transcription assay (QRIVTA) for transcriptional regulation studies
Source: Protein Cell. 2024 Oct 10;16(6):484–90. doi: 10.1093/procel/pwae054 (PMC12187118; doi:10.1093/procel/pwae054)
Supplement: pwae054_suppl_Supplementary_Figures_S1-S15_Tables_S1-S4 [file pwae054_suppl_supplementary_figures_s1-s15_tables_s1-s4.pdf]

Supplementary Materials and Methods

Preparation of plasmid templates for *in vitro* transcription

Firstly, a linear DNA fragment, mainly containing the *LEE5* promoter region (-304 to +171, defined as *LEE5p* gene) and a previously reported molecular beacon (MB1) complementary sequence (Seq_{MB1}) (Dong et al., 2018), was chemically synthesized and amplified by polymerase chain reaction (PCR). To generate the plasmid templates, a 47-bp *E. coli rrnB* T_1 terminator (*rrnB*T_1_47) sequence was attached to the 3' end of the linear DNA fragment through PCR. Meanwhile, an 1870-bp fragment of pUC19 plasmid containing the replication origin element (ori) and the antibiotic resistance gene (*AmpR*) was amplified by PCR, with a *Hin*d III restriction site in the downstream region (~80 bp) of ori and a *Nde* I restriction site in the upstream region (~50 bp) of *AmpR* gene, which was named as pUC19s. Then, the DNA fragment composed of *LEE5p*, Seq_{MB1}, and *rrnB*T_1_47 was ligated with the pUC19s through the *Hin*d III and *Nde* I restriction sites, to form the *trans*-pLEE5p or *cis*-pLEE5p template, in which *LEE5p* has a divergent or tandem orientation relative to the *AmpR*, respectively (Fig. S2A).

All the subsequent modifications on these plasmid templates were made through homologous recombination, with a Mut Express II Fast Mutagenesis Kit (Vazyme). For the nicked plasmid preparation, the recognition sequence of a nicking endonuclease Nt.*Bsp*Q I was introduced in the region between the ori and the *AmpR* gene (Fig. S2A). For the fluorescent light-up RNA aptamer-based detection strategy, Seq_{MB1} was substituted with the sequences of Spinach (Jensen et al., 2023) or iSpinach (Qin et al., 2022), respectively (Table S3 and Fig. S4A). These RNA aptamers can specifically bind to the non-fluorescent dye DFHBI in the reaction sample, causing the dye to emit fluorescence, thereby allowing for real-time detection.

To examine the termination efficiency of different transcription terminators on the *LEE5p* gene, the iSpinach sequence was inserted downstream of the terminator under investigation (Fig. S5A). The termination sequence was then changed accordingly. The degree of transcription read-through could be quantitatively evaluated by the ratio between fluorescence intensities detected at 120 min from plasmid templates with the iSpinach sequence positioned downstream (Fig. S5A) and upstream of the terminator (Fig. S4A). The termination efficiency could be subsequently determined by deducting the read-through percentage from 100%.

Similarly, the transcription efficiency of the *AmpR* gene was detected by inserting the iSpinach sequence into its downstream region (Fig. S6). To terminate the transcription of the *AmpR* gene, the *rrnBT*1_47 terminator was inserted between the *AmpR* gene and the iSpinach sequence (Fig. S6A, left) and the terminator efficiency was evaluated by detecting the fluorescence from iSpinach/DFHBI (Fig. S6B). The iSpinach sequence was also inserted immediately upstream of the *LEE5p* gene to detect the transcription in this region (Fig. S6A, right).

To construct the new *cis*-pLEE5p (*cis*-pLEE5pN) template (Fig. 1D), the *rrnBT*1_47 terminator downstream of the *LEE5p* gene in the *cis*-pLEE5p was replaced with a robust double terminator M13D, which is composed of the M13 central and *rrnD T*1 terminator sequences (Cambray et al., 2013), and Seq_{MB1} was substituted with $Seq_{MB1\text{-}hp}$, which introduces a hairpin structure, intended to improve the accessibility of Seq_{MB1} in the RNA structure (Fig. 1B). In addition, a *rrnBT*1_47 terminator sequence was also introduced in the downstream region of the *AmpR* gene in the *cis*-pLEE5pN, in order to efficiently terminate its transcription. The *trans*-pLEE5pN template was generated similarly (Fig. 1D). For the aptamer-based detection strategy, $Seq_{MB1\text{-}hp}$ was substituted with the iSpinach sequence. To construct the plasmid template without extra sequence for the fluorescence detection, $Seq_{MB1\text{-}hp}$ in the *cis*-pLEE5pN was deleted (Fig. S12A).

Supercoiled plasmids were extracted and purified using a HiPure Maxi Plasmid Kit (GeneZe). The nicked plasmids were generated by treating the supercoiled plasmids with the nicking endonuclease Nt.*Bsp*Q I (Fig. S9A) and the linearized *cis*-pLEE5pN templates were generated and amplified through PCR using two divergent primers around the specific sites (Fig. S8A). Both were purified with the *EasyPure*® PCR Purification Kit (TransGen Biotech). The purities of all the plasmid templates were estimated to be >90% based on agarose gel electrophoresis. Quantification of the plasmid templates was performed using a Nanodrop 2000 spectrophotometer (Thermo Scientific). The sequences of the primers and terminators are provided in Tables S1 and S2, respectively.

Expression and purification of H-NS

The coding sequence of H-NS was amplified using PCR from the genome of *E. coli* strain BL21 (DE3), and was cloned into the *Nde* I and *Xho* I restriction sites of a pET-21a expression vector, with a C-terminal His_6-tag coding sequence. The plasmid was transformed into *E. coli* BL21 (DE3) for protein expression. A single colony of the bacteria was inoculated into 50 mL

of Luria-Bertani (LB) medium containing 100 μg/mL ampicillin. After overnight incubation at 37 °C, the culture was transferred to 1 L of LB medium containing 100 μg/mL ampicillin. The protein expression was induced at OD_{600} 0.8 by adding 0.1 g/L IPTG. The cells were harvested by centrifugation after 6 h and resuspended using cell lysis buffer (50 mM Tris-HCl, 1 M NaCl, 20 mM imidazole, pH 8.0). The cells were disrupted by ultrasonication on ice with the addition of 1 mM PMSF and 1% Triton X-100, and the cell debris was removed by centrifugation. His-tagged H-NS protein was purified with a Ni-NTA affinity column (Qiagen), followed by gel filtration with a 16/600 Superdex^{TM} 200 pg column (GE Healthcare Life Sciences). The purified protein sample was stored in 20 mM Tris-HCl buffer, with 300 mM NaCl and 1 mM DTT, at pH 7.5. The purity of the protein was examined based on SDS-PAGE and the protein was quantified using a Nanodrop™ 2000.

Fluorescence-based real-time *in vitro* transcription assay

All the MBs were synthesized by Ruibiotech using 2'-O-methyl ribonucleotides to minimize nonspecific fluorescence (Marras et al., 2004), with a 5'-FAM fluorescent group and a 3'-DABCYL quencher group (Table S3). They were pre-annealed by heating at 90 °C for 5 min and slowly cooling down to 37 °C in 1× *E. coli* RNAP reaction buffer (NEB). The fluorescence was measured using a StepOnePlus™ Real-Time PCR System (Applied Biosystems) on the FAM/SYBR Green channel, with a MicroAmp Fast Optical 96-Well Reaction Plate (Applied Biosystems).

The total volume of each IVT reaction was 20 μL. Unless otherwise specified, a reaction mixture containing 10 nM transcription template, 500 nM pre-annealed MB for MB-based detection strategy or 25 μM DFHBI (Sigma-Aldrich) for aptamer-based detection strategy, 100 μg/mL BSA, 1 U/μL RNase inhibitor (Takara), and 1×*E. coli* RNAP reaction buffer (40 mM Tris-HCl, 150 mM KCl, 10 mM $MgCl_2$, 1 mM dithiothreitol, 0.01% Triton X-100™, pH 7.5) was first prepared and pre-incubated at 37 °C for 10 min, with or without H-NS. Then, the mixture was supplied with 1 U *E. coli* RNAP (NEB) and further incubated at 37 °C for 20 min. The transcription reaction was initiated with the addition of 500 μM pre-warmed rNTPs mixture (NEB). To ensure the consistency of initiation time across wells, the rNTPs mixture was added on the wall of each well of the 96-well plate, not touching the solution at the bottom. Then, the 96-well plate was covered with a MicroAmp Optical Adhesive Cover (Applied Biosystems) to prevent evaporation, followed by instantaneous centrifugation to mix rNTPs with the reaction mixture. Specifically, when transcription was initiated by the addition of the

transcription template, the order of the addition of rNTPs and the template was reversed. Once the transcription started, the 96-well plate was quickly put into the real-time PCR system pre-warmed at 37 °C, and the first fluorescence data point was measured after 1 min incubation. Subsequently, the fluorescence intensity was measured every 2 min. For each measurement, we concurrently measured the fluorescence of samples without RNA polymerase, which could not transcribe RNA, to serve as the negative control. For the MB2-based detection that does not exhibit strong specificity, the nonspecific fluorescence data were measured using modified plasmids as transcription templates, in which Seq_{MB2} was either mutated or deleted, under the same reaction condition.

Fluorescence data analysis

For the raw fluorescence data of each sample, the intensity of the first point was subtracted from the fluorescence intensities of all time points, to bring the initial fluorescence intensity to zero. Then, the fluorescence data of the negative control were subtracted from the fluorescence data of the transcription samples for each data point, in order to correct the baseline. Specifically, for the fluorescence data from the plasmid template without extra sequence using the MB2-based detection (Fig. S12), an additional subtraction of the nonspecific fluorescence data was performed. The resultant fluorescence data were then subjected to further curve fitting analysis.

Initially, the fluorescence data were fitted using an exponential decay (increase form) function (Equation S1) (Huang et al., 2022):

$$F(t) = \frac{v_0}{k}(1 - e^{-kt})$$

where $F(t)$ is the fluorescence intensity at time t, v_0 is the initial fluorescence production rate, and k is the attenuation rate constant of the fluorescence increase. The maximum fluorescence intensity equals v_0 divided by k. However, the Equation S1 did not provide a very good fit to the fluorescence data. To obtain a more precise fitting equation, the first derivatives of the fluorescence data, which reflects the fluorescence production rate at each time point, were analyzed (Fig. S10). It was found that the first derivative data of the fluorescence data could be well fitted to an equation (Fig. 2A, Equation 1) with an exponentially decaying rate $v_d(t)$ and a constant rate v_c (Fig. S10):

$$v(t) = v_d(t) + v_c = v_{d0}e^{-kt} + v_c$$

in which v_{d0} is the initial rate for $v_d(t)$, and k is the attenuation rate constant of $v_d(t)$. Therefore, the function of fluorescence intensity with respect to time (Fig. 2A, Equation 2) could be derived using indefinite integral:

$$F(t) = -\frac{v_{d0}}{k} e^{-kt} + v_c t + C$$

where C is a constant produced from the indefinite integral. The initial fluorescence production rate (v_0) can be calculated as the sum of v_{d0} and v_c. All the fluorescence data from supercoiled plasmid templates in this study can be precisely fitted with Equation 2. Consequently, it was used to accurately extract the initial rate (v_0) of fluorescence production and evaluate the transcription efficiency.

The numerical differentiation and curve fitting were all performed using the Origin software. All the reported values for the fitting parameters are the averages of three replicates, with the standard deviation presented.

References

Cambray, G., Guimaraes, J.C., Mutalik, V.K., Lam, C., Mai, Q.A., Thimmaiah, T., Carothers, J.M., Arkin, A.P., and Endy, D. (2013). Measurement and modeling of intrinsic transcription terminators. Nucleic Acids Res. 41, 5139-5148.

Dong, J., Wu, T., Xiao, Y., Chen, L., Xu, L., Li, M., and Zhao, M. (2018). Target-triggered transcription machinery for ultra-selective and sensitive fluorescence detection of nucleoside triphosphates in one minute. Biosens. Bioelectron. 100, 333-340.

Huang, Y.H., Trapp, V., Puro, O., Mäkinen, J.J., Metsä-Ketelä, M., Wahl, M.C., and Belogurov, G.A. (2022). Fluorogenic RNA aptamers to probe transcription initiation and co-transcriptional RNA folding by multi-subunit RNA polymerases. Meth. Enzymol. 675, 207-233.

Jensen, D., Ruiz Manzano, A., Rector, M., Tomko, E.J., Record, M.T., and Galburt, E.A. (2023). High-throughput, fluorescent-aptamer-based measurements of steady-state transcription rates for the Mycobacterium tuberculosis RNA polymerase. Nucleic Acids Res. 51, e99.

Marras, S.A., Gold, B., Kramer, F.R., Smith, I., and Tyagi, S. (2004). Real-time measurement of in vitro transcription. Nucleic Acids Res. 32, e72.

Qin, W., Li, L., Yang, F., Wang, S., and Yang, G.-Y. (2022). High-throughput iSpinach fluorescent aptamer-based real-time monitoring of in vitro transcription. Bioresour. Bioprocess. 9, 112.

Supplementary Tables

Table S1. Sequences of primers.

Primer	Sequence* (5'-3')	Purpose
cis_LEE5p_F	CCCAAGCTTAGTGATATCAAGGCTCTAACTG	Construction of the *cis*-pLEE5p and *trans*-pLEE5p plasmids
trans_LEE5p_F	GGAATTCCATATGAGTGATATCAAGGC	
LEE5p_R47_R1	ACTGAGCCTTTCGTTTTATTTGCTGCAGAACCAACACAC C	
cis_LEE5p_R47_R2	GGAATTCCATATGATAAAACGAAAGGCCCAGTCTTTCG ACTGAGCCTTTCGTTTTATT	
trans_LEE5p_R47_R2	CCCAAGCTTATAAAACGAAAGGCCCAGTCTTTCGACTG AGCCTTTCGTTTTATT	
Puc19s_HindIII_F	CTAAGCTTGGGGATAACGCAGGAAAGAAC	
Puc19s_NdeI_R	GATATCACTCATATGTGATAATAATGGTTTCTTAGACGT CAGG	
Mut_BspQI_F	aacgtgctcttccgttcCACTGAGCGTCAGACC	Introduction of the nicked site
Mut_BspQI_R	gaacggaagagcacgttAAGGGATTTTGGTCATGA	
Mut_Seq$_{MB2}$_F	attaagggggacgcggcgCACTACCTTCACAAACAGACGGC	Mutation or deletion of the Seq$_{MB2}$
Mut_Seq$_{MB2}$_R	cgccgcgtcccccttaatAATGATTGCCATTTACATTATTACCAA	
Del_Seq$_{MB2}$_F	ggcaatcattcactacctTCACAAACAGACGGC	
Del_Seq$_{MB2}$_R	aggtagtgaatgattgccATTTACATTATTACCAA	
Spinach_F	acaaacagacggcgcctcgagGACGCGACCGAAATGGTG	Substitution of Seq$_{MB1}$ with the aptamer sequences in *cis*-pLEE5p plasmid
Spinach_R	ctttcgttttatttgctgcagGACGCGACCAGTTACGGAGC	
iSpinach_F	acaaacagacggcgcctcgagGCGACTACGGTGAGGGTCG	
iSpinach_R	ctttcgttttatttgctgcagGCGACTACGGAGCCCACAC	
T_plasmid_F	gtggcacttttcggggaa	Insertion of the iSpinach sequence downstream of the terminator of *LEE5p*
T_plasmid_R	ctgacgtctaagaaaccattATTATCAC	
T_iSpinach_F	aatggtttcttagacgtcagGCGACTACGGTGAGGGTCG	
T_iSpinach_R	atttccccgaaaagtgccacGCGACTACGGAGCCCACAC	
AmpR_plasmid_F	TAGATTGATTTAAAACTTCATTTTTAATTTAA	Insertion of the iSpinach sequence downstream of the *AmpR*
AmpR_plasmid_R	AAGTATATATGAGTAAACTTGGTCTGACAGTT	
AmpR_iSpinach_F	aagtttactcatatatacttGCGACTACGGTGAGGGTCG	
AmpR_iSpinach_R	tgaagttttaaatcaatctaGCGACTACGGAGCCCACAC	
AmpR_T_plasmid_F	TACTCATATATACTTGCGACTACGGTGAGG	Insertion of the *rrnBT*$_1$_47 terminator between the *AmpR* and the iSpinach sequence
AmpR_T_plasmid_R	AACTTGGTCTGACAGTTACCAATGCT	
AmpR_T_iSpinach_F	ggtaactgtcagaccaagttCAAATAAAACGAAAGGCTCAG-TCGAAAGACTGGG	
AmpR_T_iSpinach_R	gtcgcaagtatatatgagtaATAAAACGAAAGGCCCAG-TCTTTCGACTGAGCC	

iSpinach_LEE5p_F<br>iSpinach_LEE5p_R	ttctttcctgcgttatccccGCGACTACGGTGAGGGTC<br>agccttgatatcactAAGCTTGCGACTACGGAGCCCAC	Insertion of the iSpinach sequence immediately upstream of the *LEE5p*
Seq_{MB1}_to_Seq_{MB1-hp}_F<br>Seq_{MB1}_to_Seq_{MB1-hp}_R	acaaacagacggcgcCTCGAGGCGGGCCCAAAAAAAATGTT-GTGGTGTGTTGGTTAAA<br>ttatcagcttgctttCTGCAGGCGGGCCCTTTTTTTTAAC-CAACACACCACAACATTT	Substitution of Seq_{MB1} with Seq_{MB1-hp}
Seq_{MB1-hp}_to_iSpinach_F<br>Seq_{MB1-hp}_to_iSpinach_R	acaaacagacggcgcCTCGAGGCGACTACGGTGAGGGTC<br>ttatcagcttgctttCTGCAGGCGACTACGGAGCCCAC	Substitution of Seq_{MB1-hp} with the iSpinach sequence in the *cis*-pLEE5pN plasmid
Del_Seq_{MB1-hp}_F<br>Del_Seq_{MB1-hp}_R	ctcgagaaagcaagctgaTAAACCGATACAA<br>tcagcttgctttctcgagGCGCCGTCTGTTTG	Deletion of Seq_{MB1-hp} in the *cis*-pLEE5pN plasmid
Linear1_pLEE5pN_F<br>Linear1_pLEE5pN_R<br>Linear2_pLEE5pN_F<br>Linear2_pLEE5pN_R<br>Linear3_pLEE5pN_F<br>Linear3_pLEE5pN_R	GTTCCACTGAGCGTCAGACCCCG<br>GGAAGAGCACGTTAAGGGATTTTGGTCATGAG<br>CATATGTGATAATAATGGTTTCTTAGACGTCAGGTGG<br>CGGGAGAGTGTTCACCGAC<br>AGTGATATCAAGGCTCTAACTGAGAAGGCG<br>AAGCTTGGGGATAACGCAGGAAAG	Linearization of the *cis*-pLEE5pN plasmids

* Lowercase letters indicate the homology arm for the homologous recombination.

Table S2. Sequences of terminators.

Terminator	Sequence (5’-3’)
rrnBT$_1$_47	CAAATAAAACGAAAGGCTCAGTCGAAAGACTGGGCCTTTCGTTTTAT
rrnBT$_1$_91	GGGAACTGCCAGGCATCAAATAAAACGAAAGGCTCAGTCGAAAGACTGGGCC TTTCGTTTTATCTGTTGTTTGTCGGTGAACGCTCTCCTG
BBaU10	AAAAAAAAACCCCGCCCCTGACAGGGCGGGGTTTTTTTTTT
ECK	TTCAGCCAAAAAACTTAAGACCGCCGGTCTTGTCCACTACCTTGCAGTAATGC GGTGGACAGGATCGGCGGTTTTCTTTTCTCTTCTCAA
L3S2P21	CTCGGTACCAAATTCCAGAAAAGAGGCCTCCCGAAAGGGGGGCCTTTTTTCGT TTTGGTCC
RBT	CAAATAAAACGAAAGGCTCAGTCGAAAGACTGGGCCTTTCGTTTTATAAAAAA AAACCCCGCCCCTGACAGGGCGGGGTTTTTTTTTT
M13D	AAAGCAAGCTGATAAACCGATACAATTAAAGGCTCCTTTTGGAGCCTTTTTTTT TGGAGATTTTCAACATGAAAAAATTATTATTTGATGATCAGATAGCGGCGGGG AACTGCCAGACATCAAATAAAACAAAAGGCTCAGTCGGAAGACTGGGCCTTTT GTTTTATCTGTTGTTTGTCGGTGAACACTCTCCCG

Table S3. Sequences of molecular beacons and aptamers.

MB/Aptamer	Sequence* (5’-3’)
MB1	(FAM)-CCUGGCAACCAACACACCACAACAUGCCAGG-(DABCYL)
MB2	(FAM)-CCUGGCAGCGGCGCAGGGGGAAUUAUGCCAGG-(DABCYL)
MB1s	(FAM)-CCUGCAACCAACACACCACAACAUGCAGG-(DABCYL)
Spinach	GACGCGACCGAAATGGTGAAGGACGGGTCCAGTGCTTCGGCACTGTTGAGTAGAG TGTGAGCTCCGTAACTGGTCGCGTC
iSpinach	GCGACTACGGTGAGGGTCGGGTCCAGTAGCTTCGGCTACTGTTGAGTAGAGTGTG GGCTCCGTAGTCGC

*Underlined bases represent the loop region of MB.

Table S4. Kinetic parameters for the transcription repression ability of H-NS from QRIVTA with different detection strategies.

Detection strategy	H-NS (μM)	v_0 (a.u./min)	v_{d0} (a.u./min)	k (/min)	v_c (a.u./min)	Relative transcription (%)
MB1s	0	567 ± 14	464 ± 14	0.052 ± 0.002	103 ± 1	100 ± 3
	1.0	519 ± 13	421 ± 11	0.049 ± 0.002	98 ± 2	91 ± 2
	1.5	246 ± 6	181 ± 4	0.049 ± 0.001	65 ± 2	43 ± 1
	2.0	146 ± 15	104 ±13	0.064 ± 0.004	42 ± 2	26 ± 3
	3.0	32 ± 2	20 ± 3	0.022 ± 0.006	12 ± 2	6 ± 0
iSpinach	0	313 ± 2	260 ± 2	0.057 ± 0.003	53 ± 1	100 ± 0
	1.0	284 ± 5	233 ± 4	0.056 ± 0.003	51 ± 2	91 ± 2
	1.5	143 ± 11	111 ±11	0.051 ± 0.004	31 ± 0	46 ± 3
	2.0	72 ± 11	54 ± 8	0.056 ± 0.009	18 ± 2	23 ± 3
	3.0	28 ± 1	20 ± 2	0.028 ± 0.005	7 ± 1	9 ± 0
MB2	0	205 ± 1	187 ± 2	0.027 ± 0.002	17 ± 2	100 ± 0
	1.0	181 ± 4	171 ± 3	0.022 ± 0.002	10 ± 4	88 ± 2
	1.5	98 ± 8	79 ± 8	0.065 ± 0.003	19 ± 2	48 ± 4
	2.0	60 ± 7	50 ± 6	0.056 ± 0.019	11 ± 3	30 ± 3
	3.0	18 ± 4	15 ± 6	0.025 ± 0.011	3 ± 3	9 ± 2

Supplementary Figures

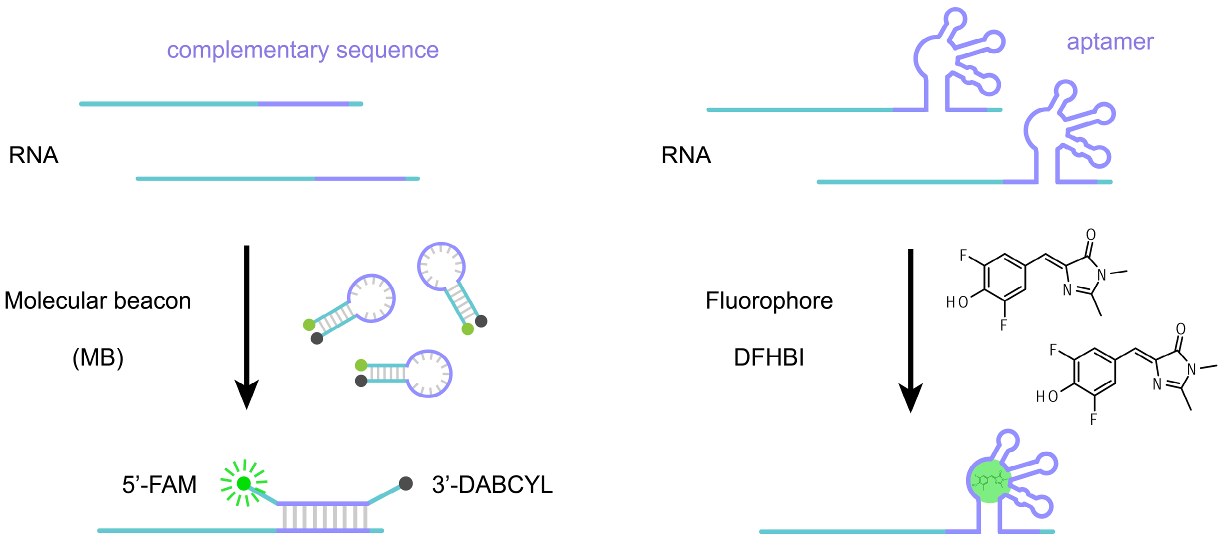

Figure S1. Illustrations of the molecular beacon- (MB) and RNA aptamer-based detection strategies.

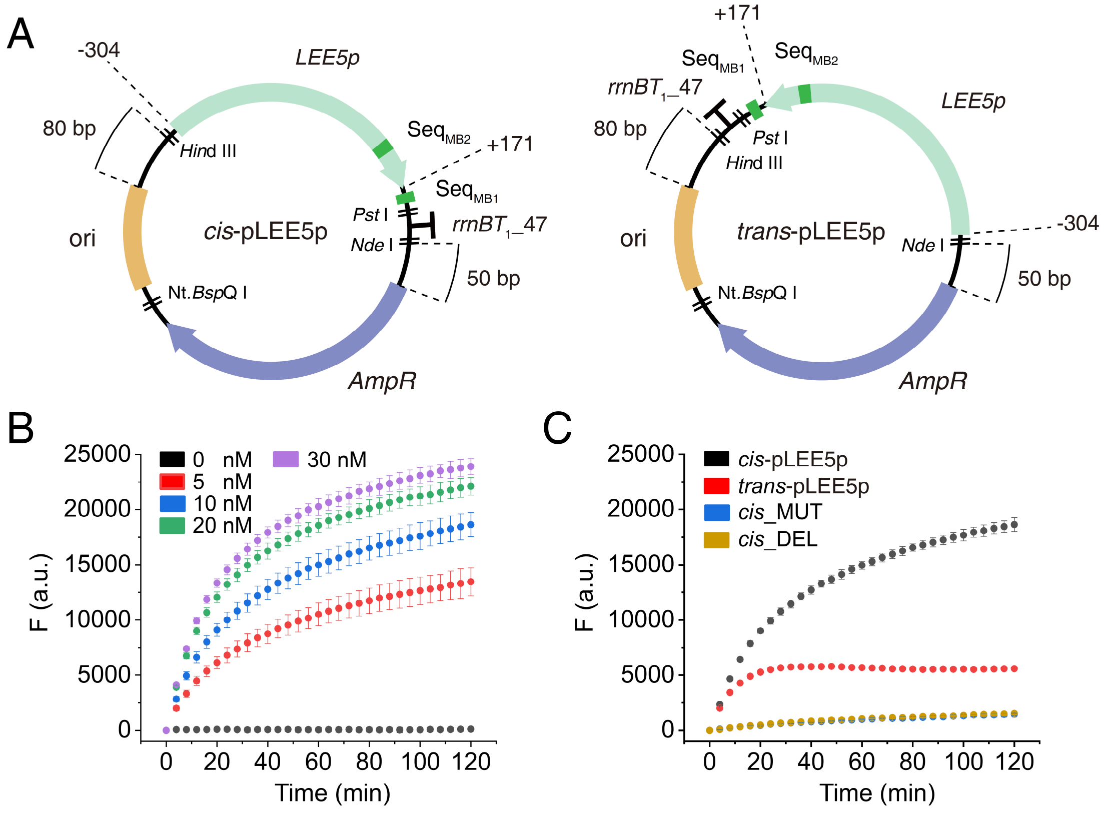

Figure S2. Comparison of supercoiled *cis*-pLEE5p and *trans*-pLEE5p templates. (A) Plasmid design of *cis*-pLEE5p and *trans*-pLEE5p. (B) Time courses of the fluorescence production from RT-IVT assays with supercoiled *cis*-pLEE5p template at indicated concentrations, using MB2-based detection at 37 °C. (C) Time courses of the fluorescence production from RT-IVT assays with 10 nM supercoiled *cis*-pLEE5p (black), *trans*-pLEE5p (red), *cis*_MUT (blue), and *cis*_DEL (orange) templates, using MB2-based detection at 37 °C. Seq_{MB2} was either mutated or deleted in the *cis*-pLEE5p to construct the *cis*_MUT or *cis*_DEL plasmid template, respectively. Each data point is the average of three replicates, with the error bar indicating the standard deviation.

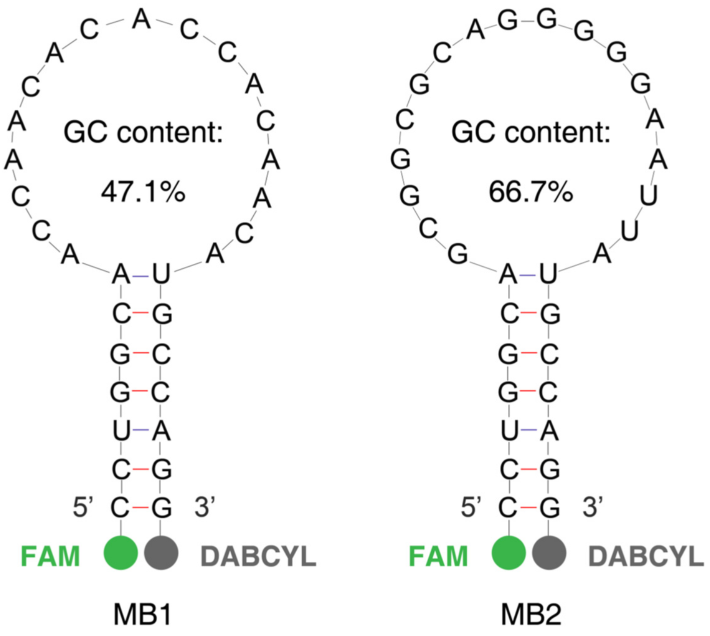

Figure S3. Designs of molecular beacon 1 (MB1) and molecular beacon 2 (MB2).

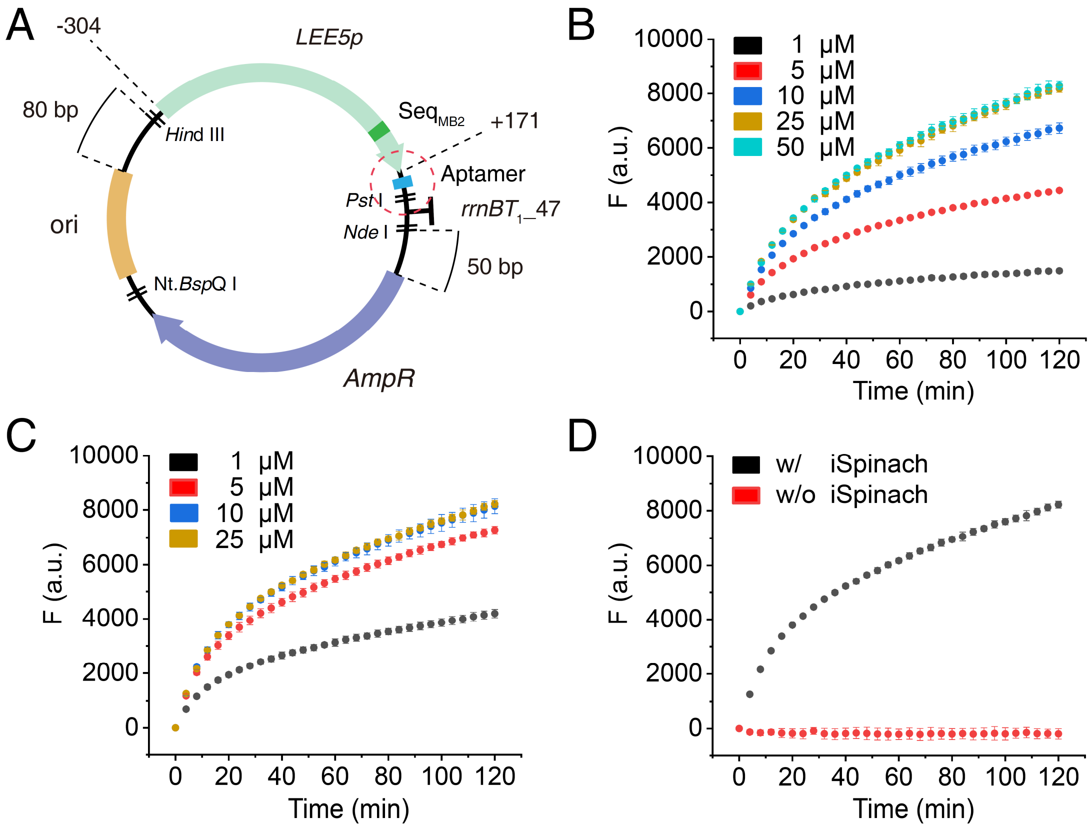

Figure S4. RT-IVT assays using aptamer-based detection with supercoiled plasmid template. (A) Plasmid design for the aptamer- (Spinach- or iSpinach-) based detection. Time courses of the fluorescence production from RT-IVT assays using Spinach- (B) or iSpinach- (C) based detection with 10 nM supercoiled plasmid template, in the presence of DFHBI at indicated concentrations, at 37 °C. iSpinach exhibits higher fluorescence intensity than Spinach when the concentration of DFHBI is relatively low (≤10 μM), while both aptamers demonstrate comparable fluorescence intensities at higher DFHBI concentrations (≥25 μM), presumably due to the over-saturation of DFHBI. (D) Time courses of the fluorescence production from RT-IVT assays using iSpinach-based detection in the presence of 25 μM DFHBI at 37 °C, with 10 nM supercoiled plasmid templates, either with or without the iSpinach sequence. Each data point is the average of three replicates, with the error bar indicating the standard deviation.

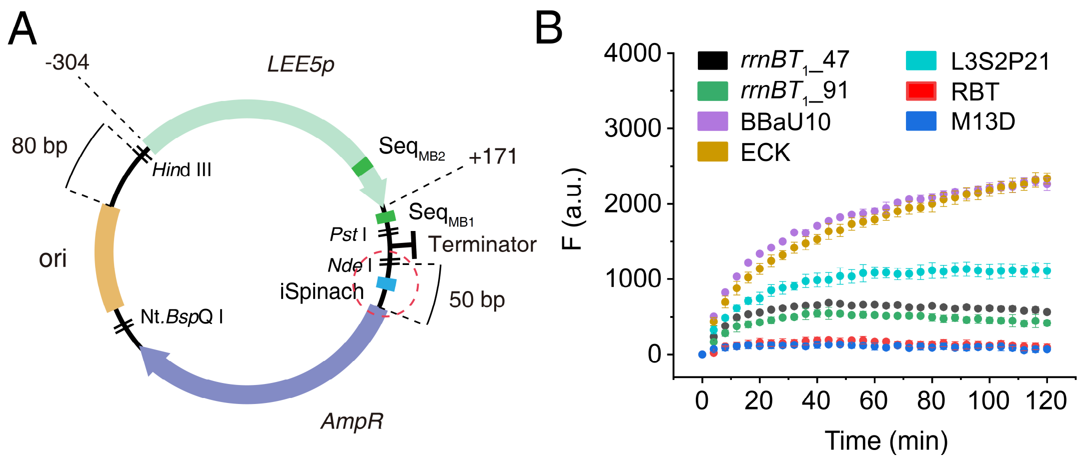

Figure S5. Evaluation of the termination efficiency of transcription terminators. (A) Plasmid design for termination efficiency measurements. The iSpinach sequence is positioned downstream of the target terminator for the evaluation of transcription read-through. (B) Time courses of the fluorescence production from RT-IVT assays for the indicated terminators, using iSpinach-based detection, with 10 nM supercoiled plasmid template. The 91-bp "*rrn*B T1" (*rrnBT*$_1$_91), "M13 central + *rrn*D T1" (M13D), and BBa_B1006 U10 (BBaU10), as well as ECK120029600 (ECK) and L3S2P21 are previously reported terminators with high efficiency. The 47-bp "*rrn*B T1" (*rrnBT*$_1$_47) and "*rrnBT*$_1$_47 + BBaU10" (RBT) are newly designed terminators in this study. All the experiments were performed in the presence of 25 μM DFHBI at 37 °C. Each data point is the average of three replicates, with the error bar indicating the standard deviation.

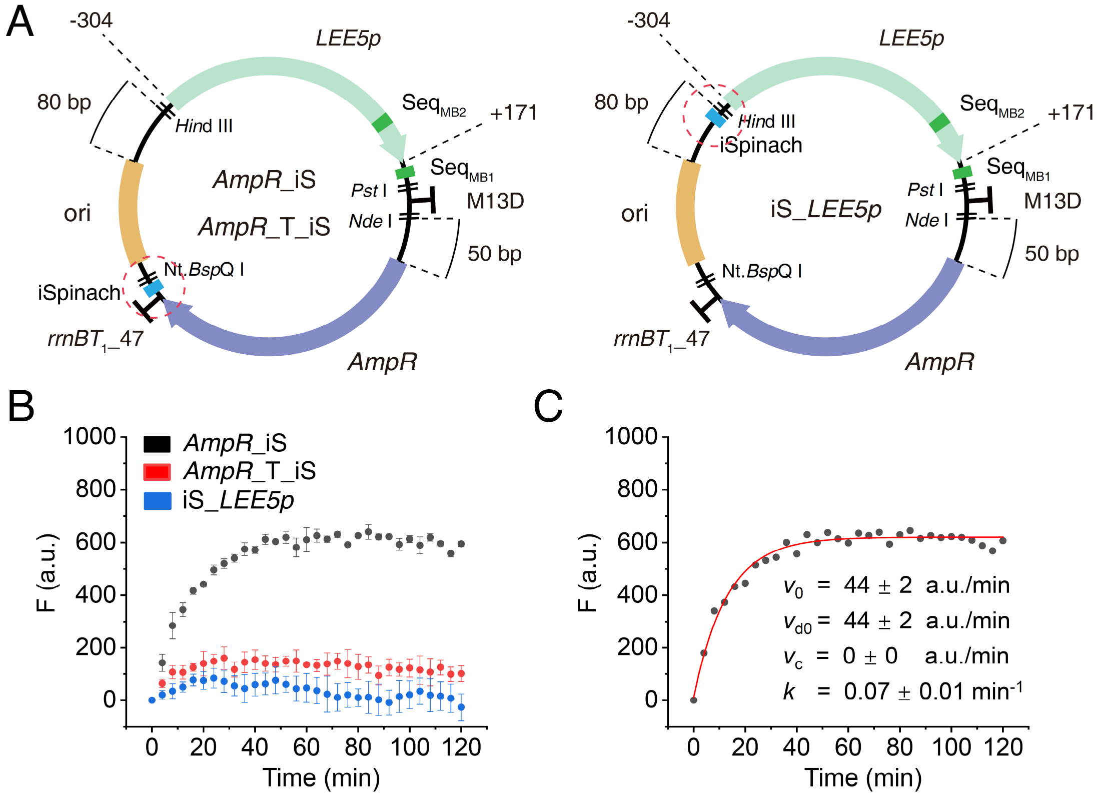

Figure S6. Transcription termination of *AmpR* gene. (A) Plasmid designs for the detection of transcription read-through of the *AmpR* gene, with the iSpinach sequence positioned downstream of the *AmpR* (*AmpR*_iS and *AmpR*_T_iS) or upstream of the *LEE5p* (iS_*LEE5p*). *AmpR*_T_iS was generated by inserting the *rrnBT*1_47 terminator between *AmpR* gene and iSpinach. (B) Time courses of the fluorescence production from RT-IVT assays using iSpinach-based detection in the presence of 25 µM DFHBI at 37 °C, with 10 nM supercoiled plasmid template as indicated. The low fluorescence observed with the *AmpR*_T_iS plasmid template indicates effective termination of *AmpR* transcription by the *rrnBT*1_47 terminator. Similarly, the low fluorescence observed with the iS_*LEE5p* plasmid template confirms that there is essentially no leaky transcription that would interfere with the transcription of *LEE5p* with this plasmid construct. Each data point is the average of three replicates, with the error bar indicating the standard deviation. (C) The representative fitting curve (red line) for fluorescence data from the *AmpR*_iS plasmid template using Equation 2. Fitting parameters are shown.

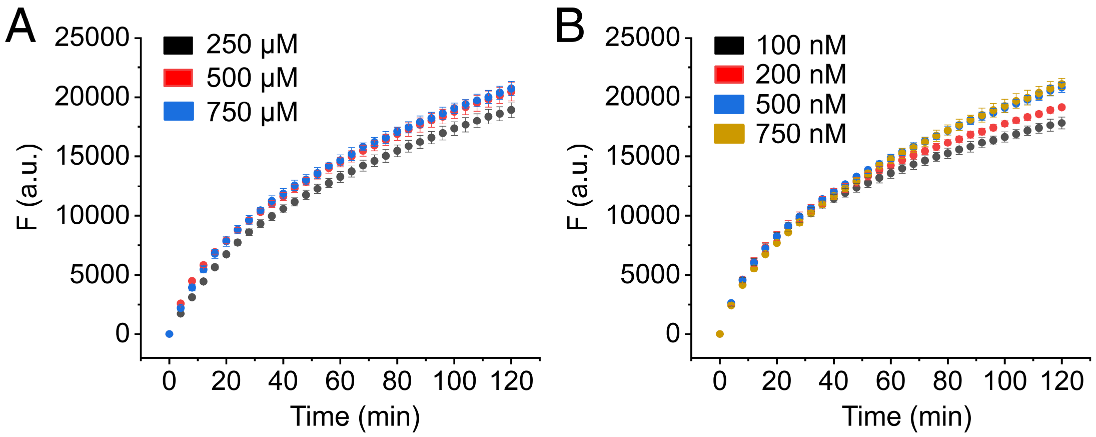

Figure S7. Excessive rNTPs and MB used in the RT-IVT assay. Time courses of the fluorescence production from RT-IVT assays using MB1s-based detection with 10 nM supercoiled *cis*-pLEE5pN template at 37 °C, in the presence of rNTPs (A) or MB1s (B) at indicated concentrations. Increasing the rNTPs concentration from 0.25 mM to 0.5 mM slightly increases the transcriptional fluorescence intensity, whereas no additional enhancement is observed after a further increase to 0.75 mM, within the 120 min experiment time. Similarly, a plateau is reached with respect to fluorescence intensity enhancement when the MB1s concentration is increased to 500 nM. Each data point is the average of three replicates, with the error bar indicating the standard deviation.

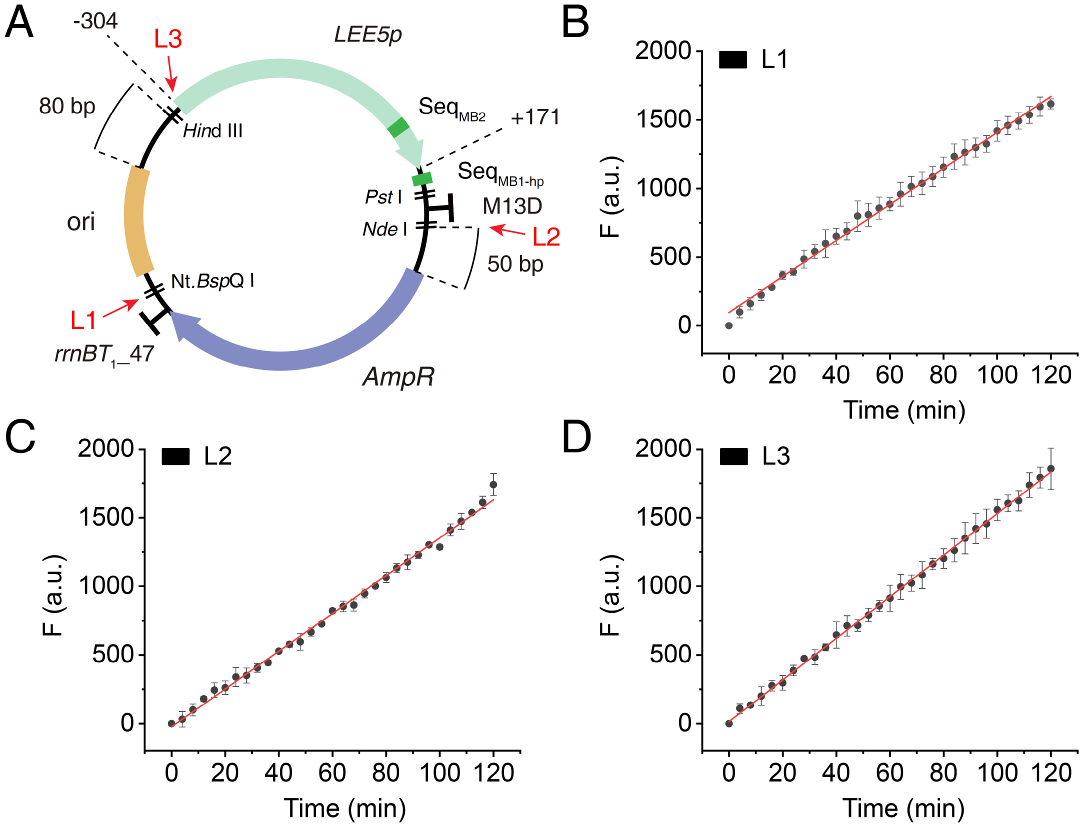

Figure S8. RT-IVT assays with linearized *cis*-pLEE5pN templates. (A) Linearized sites (L1, L2, L3) on the *cis*-pLEE5pN are indicated by red arrows. Time courses of the fluorescence production from RT-IVT assays using MB1s-based detection with 10 nM linearized plasmid templates at L1 (B), L2 (C), or L3 (D) sites at 37 °C, with the linear regression lines shown in red. Each data point is the average of three replicates, with the error bar indicating the standard deviation.

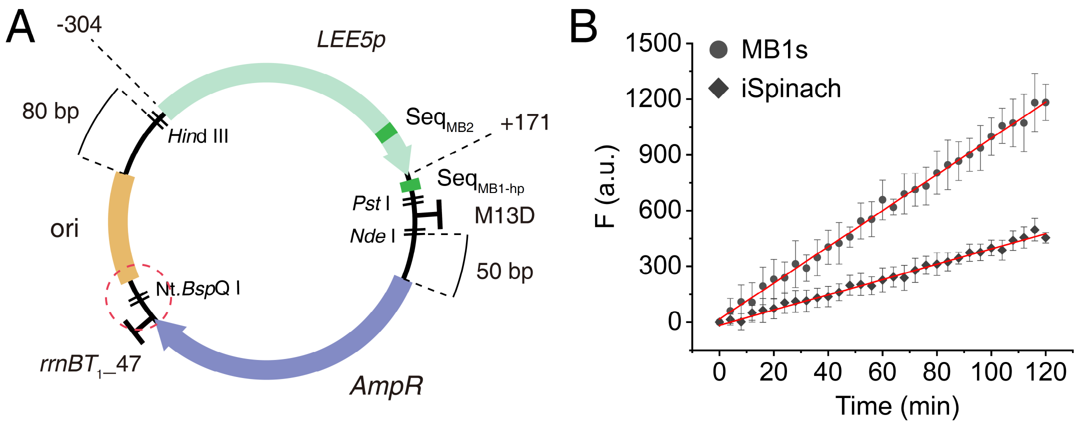

Figure S9. RT- IVT assays with nicked *cis*-pLEE5pN template. (A) The nicked plasmid was prepared by treating the supercoiled *cis*-pLEE5pN with the Nt.*Bsp*Q I restriction enzyme. (B) Time courses of the fluorescence production from RT-IVT assays using MB1s- (circle) or iSpinach- (diamond) based detection with 10 nM nicked plasmid template at 37 °C, with the linear regression lines shown in red. Each data point is the average of three replicates, with the error bar indicating the standard deviation.

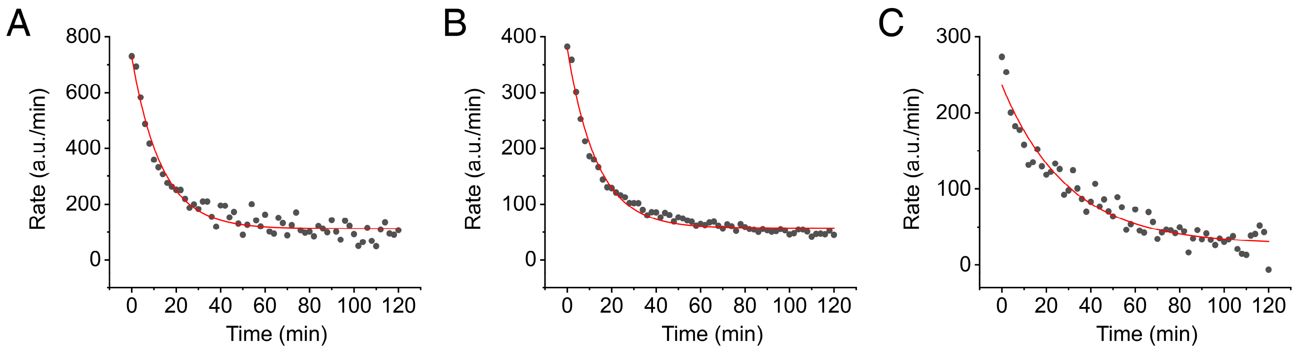

Figure S10. First derivative analysis of fluorescence data from RT-IVT assays with supercoiled plasmid templates. The representative first derivative data of fluorescence data using MB1s- (A), iSpinach- (B), or MB2- (C) based detection, with the fitting curves using Equation 1 shown in red.

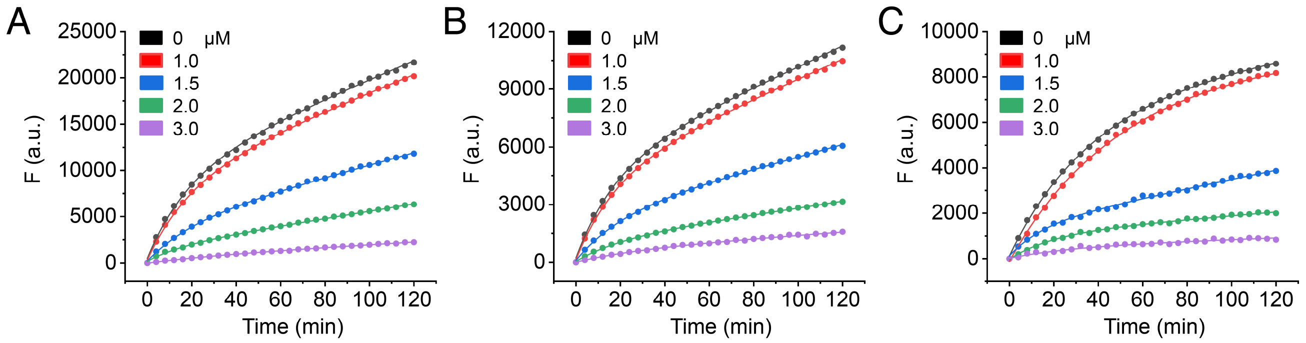

Figure S11. Fitting curves using Equation 2 for fluorescence data from supercoiled plasmid templates. The representative fitting curves (lines) of fluorescence data (dots) using MB1s- (A), iSpinach- (B), or MB2- (C) based detection, in the absence or presence of H-NS at indicated concentrations.

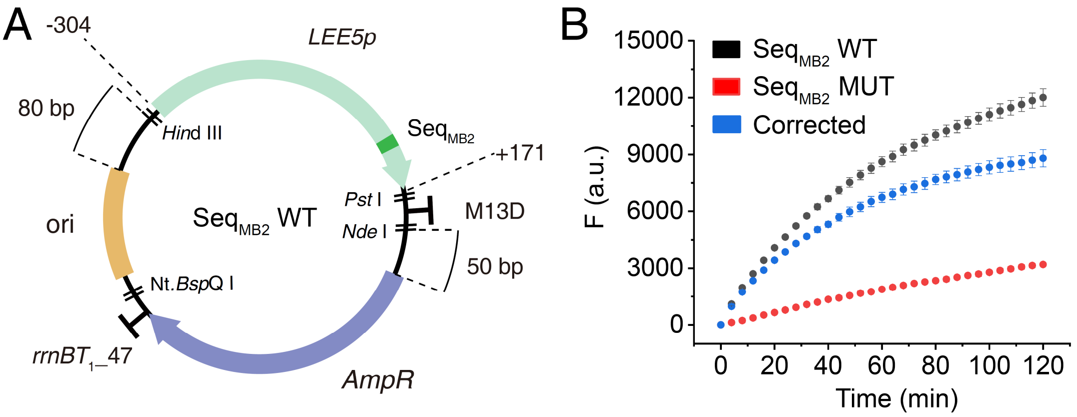

Figure S12. RT-IVT assays using MB2-based detection with supercoiled plasmid template devoid of extra sequence for the fluorescence detection. (A) Plasmid design for MB2-based detection without the introduction of extra sequence. (B) Time courses of the fluorescence production from RT-IVT assays using MB2-based detection with 10 nM supercoiled plasmid (Seq_{MB2}WT) template shown in (A). The Seq_{MB2}MUT plasmid with mutated Seq_{MB2} is used as a control for nonspecific fluorescence detection. The corrected specific fluorescence data are generated by subtracting the nonspecific fluorescence data from the raw fluorescence data (Seq_{MB2}WT plasmid). Each data point is the average of three replicates, with the error bar indicating the standard deviation.

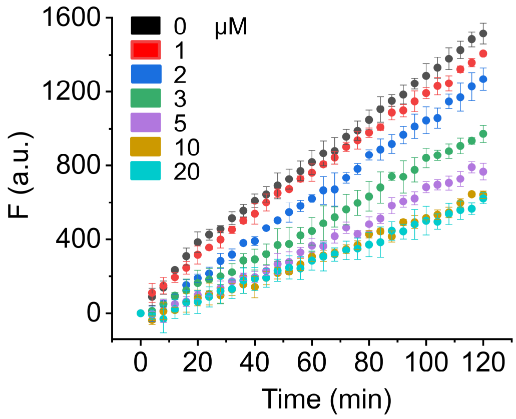

Figure S13. The transcription repression ability of H-NS on linearized plasmid template. Time courses of the fluorescence production from QRIVTA using MB1s-based detection with 10 nM linearized *cis*-pLEE5pN (L1) template, in the absence or presence of H-NS at indicated concentrations, at 37 °C. Each data point is the average of three replicates, with the error bar indicating the standard deviation.

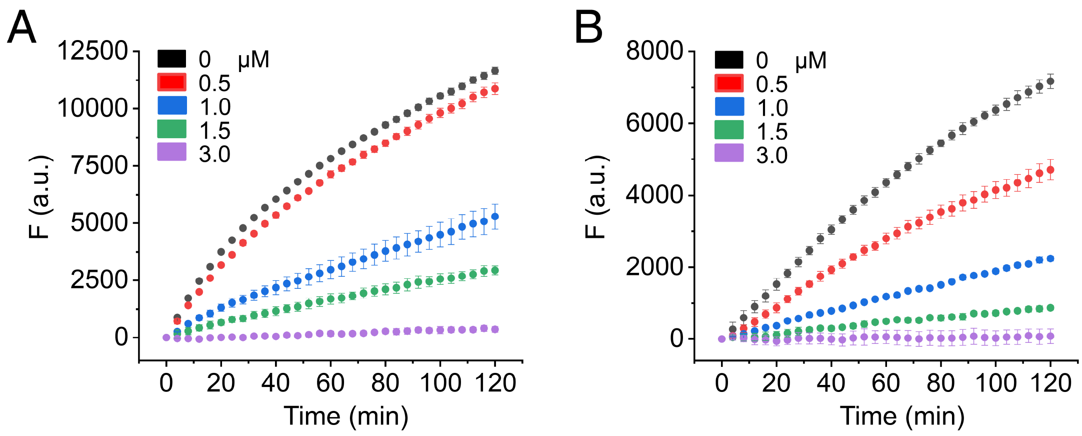

Figure S14. Effect of temperature on the transcription repression ability of H-NS on supercoiled *cis*-pLEE5pN template. Time courses of the fluorescence production from QRIVTA using MB1s-based detection with 10 nM supercoiled *cis*-pLEE5pN template in the absence or presence of H-NS at indicated concentrations, at 30 °C (A) or 25 °C (B).

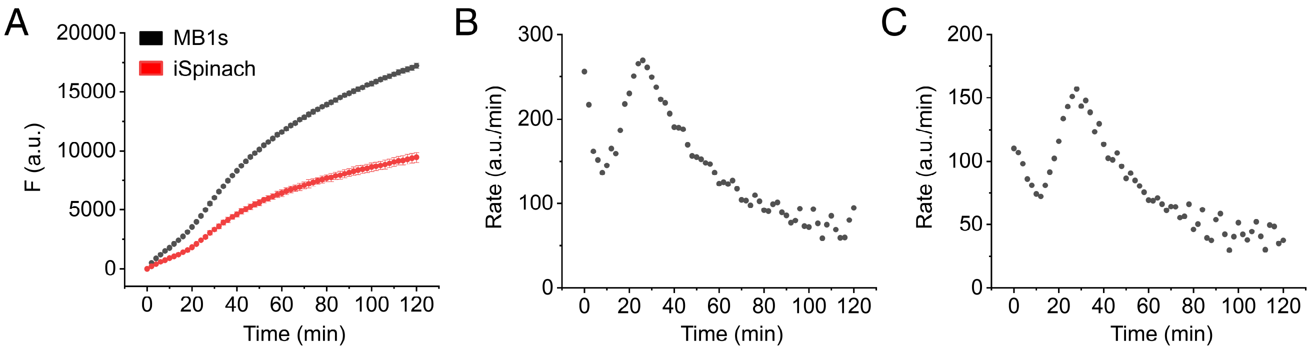

Figure S15. RT-IVT assays initiated by the addition of supercoiled plasmid template. (A) Time courses of the fluorescence production from RT-IVT assays initiated by adding 10 nM supercoiled *cis*-pLEE5pN template using MB1s- (black) based detection at 37 °C. For iSpinach-based detection (red), $Seq_{MB1\text{-}hp}$ in the *cis*-pLEE5pN is substituted with the iSpinach sequence. Each data point is the average of three replicates, with the error bar indicating the standard deviation. The representative first derivative data of fluorescence data from the MB1s- (B) and iSpinach- (C) based detection are shown.

Guidelines for QRIVTA

To accurately reflect the transcription process and transcriptional regulation, the transcription kinetics must be taken into account, which largely depends on quantitative analysis of the data from IVT assays with real-time detection. All of the results in this study demonstrate the versatility and adaptability of QRIVTA for quantitative studies of transcriptional regulation. The QRIVTA enables the quantitative analysis and comparison of different intrinsic and extrinsic factors that influence transcription and transcriptional regulation, thereby aiding in a deeper comprehension of the underlying mechanisms in these processes. It is worth emphasizing that variations in the IVT experimental procedure are very likely to affect the kinetics of the fluorescence production, which may result in poor data repeatability, reproducibility, and comparability. Consequently, it is crucial to standardize the fluorescence-based real-time IVT assay procedure, including the aspects of assay design, experimental operation, and data analysis. Therefore, we propose the following guidelines for carrying out QRIVTA for the study of transcriptional regulation:

Plasmid template design: Given the presence of additional gene transcription on the plasmid template aside from the target gene, it is necessary to examine the transcription read-through of each gene on the template, to avoid any interference on the transcription of the target gene. We have revealed significant differences in the termination efficiency of certain terminators when compared *in vitro* and *in vivo*, which may be attributed to variations in sequence context and reaction conditions. Consequently, it is important to evaluate the efficiency of specific terminators under targeted experimental conditions, and the termination efficiency can be potentially enhanced through the strategy of terminator concatenation.

Fluorescence detection strategy: The aptamer-based detection strategy possesses considerable specificity and requires minimal design and optimization, making it a versatile tool for a range of tests and preliminary studies. The MB-based detection strategy could directly detect the transcription without the introduction of any extra sequences, and MB could be easily manipulated and optimized to achieve superior detection sensitivity and simultaneous detection of multiple targets. Therefore, it is suitable for assays requiring high-sensitivity detection, serving as a custom solution under specialized research contexts. The sensitivity of aptamer-based detection strategy could also be enhanced by using newly developed fluorescent light-up RNA aptamers and fluorophore ligands, such as Pepper/HBC530 (Chen et al., 2019) and Okra/ACE (Zuo et al., 2024).

To efficiently carry out the optimization of the MB-based detection strategy, several key points should be taken into account. The foremost factor is the design of the MB. Typically, MBs synthesized from 2'-O-methyl ribonucleotides are designed to encompass a stem region consisting of 5 to 6 base pairs, along with a loop region comprising 15 to 30 base pairs (Catrina et al., 2012). In addition, it is advisable for the loop region of MB to maintain a GC content of 30-60% and to avoid sequences with more than three consecutive G/C bases, to prevent nonspecific binding. The length and GC content of the stem and loop region should be adjusted to optimize the sensitivity and specificity of QRIVTA, in accordance with the specific system studied. Another crucial factor is the accessibility of the complementary sequence on the RNA. When selecting complementary sequences for the loop region, the secondary structure of the target RNA should be analyzed using software such as Mfold (Zuker, 2003), and it is recommended to choose the regions with minimal secondary structures as the complementary sequences for the design of MBs. If the appropriate regions are unobtainable, the accessibility of complementary regions on the RNA can be enhanced by the insertion of extra sequences with defined structures, such as the hairpin structures adopted in this study.

Sensitivity and specificity tests: Prior to the formal experiments, the sensitivity and specificity of fluorescence detection strategies should be carefully tested to ensure they meet the requirements of the target experiments. The detection sensitivity could be directly indicated by fluorescence intensity, which ought to be significantly higher than the baseline fluctuation deviation. Higher detection sensitivity is particularly beneficial for conducting QRIVTA with a real-time PCR thermocycler. Nonspecific fluorescence can be measured using a modified plasmid as the transcription template, in which the target sequence for fluorescent detection is mutated or deleted. In cases where the introduction of extra sequences is undesirable and the designed MB only exhibits moderate specificity, the nonspecific fluorescence data can be subtracted from the raw fluorescence data to obtain the specific fluorescence data.

Experimental operation: In preparation for QRIVTA, the samples of transcription factors, transcription template, MB or DFHBI, RNA polymerase inhibitor, and RNAP reaction buffer should be first thoroughly mixed. These mixtures are then pre-incubated at the experimental temperature to facilitate the binding-dissociation equilibrium between the transcription factors and the DNA template. The RNAP reaction buffer can also be adjusted for special study systems, such as examining the effects of metal ions on the transcriptional regulation of certain transcription factors. Subsequently, the RNAP is introduced, followed by further incubation at

the experimental temperature to re-establish the equilibrium within the system. Then, the pre-warmed rNTPs are added on the wall of each reaction well to avoid direct contact with the reaction mixture at the bottom. To minimize evaporation, especially at high experimental temperatures that could markedly influence the fluorescence readings, an optical adhesive cover should be securely covered on the reaction plate. Finally, instantaneous centrifugation is performed at the experimental temperature to homogenize the rNTPs with the samples. Once the transcription starts, the reactions should be quickly put into the pre-warmed real-time PCR system for fluorescence detection. It is noteworthy that the concentration of rNTPs and fluorophore-containing reagents should be in vast excess for IVT assays, in order to minimize their effects on the transcription rate due to their consumptions during the experimental time.

For each measurement, a corresponding negative control, which is devoid of RNA polymerase, should be set up concurrently to monitor the fluorescence baseline. All the samples should be pre-warmed to the experimental temperature before the fluorescence detection, in order to avoid fluorescence perturbation at early time points. Due to possible batch-to-batch variations, especially concerning the RNAP enzymatic activity, it is preferable to carry out assays with the same batch of enzyme under identical experimental conditions, for better data repeatability, reproducibility, and comparability. Otherwise, it is necessary to calibrate the activity of the RNAP enzyme from different batches.

Data analysis: Firstly, preprocessing of the raw fluorescence data is required, which involves the subtraction of the first fluorescence intensity from all the data points to bring the initial fluorescence intensity to zero, followed by a baseline correction through the subtraction of the fluorescence data of the negative control. In the cases of the presence of nonspecific fluorescence, it is also necessary to subtract the nonspecific fluorescence control data from the raw data. Subsequently, these fluorescence data are subject to curve fitting using Equation 2 to extract kinetic parameters of fluorescence production. The curve fitting procedure can be easily performed using the nonlinear curve fitting functions available in a series of software, such as Origin, GraphPad Prism, SigmaPlot, etc.

The fluorescence production rate of the RT-IVT data is composed of a time-dependent exponentially decaying rate $v_d(t)$ and a time-independent constant rate v_c, with an attenuation rate constant k for $v_d(t)$. The initial fluorescence production rate v_0 is the sum of the initial value v_{d0} for the decaying rate and constant rate v_c, which is the maximum fluorescence production rate during the RT-IVT assay and can be used to reflect the maximal transcription rate and the

overall transcription efficiency. Although we got similar transcription repression abilities for H-NS using the measure of $F(t)$ at a given time, the results vary for fluorescence data at different time points. As the initial fluorescence production rate v_0 has a clear definition and is also a commonly used parameter in traditional enzymology studies, it is more reasonable and standard to use v_0 for the overall transcription efficiency evaluation. However, it is interesting to find that a constant rate term v_c is needed to produce a better fit of the fluorescence curves with the supercoiled plasmid template. We suspected that the v_c may arise from a mechanism that can partially alleviate the causes for the attenuation, such as spontaneous supercoil diffusion and relaxation (Deng et al., 2004; Moulin et al., 2005; Sobetzko, 2016). Both the parameters k, v_{d0}, and v_c are needed for the precise quantitative analysis of the fluorescence production kinetics, and further investigations are required to reveal the molecular mechanisms underlying these parameters.

References

Catrina, I.E., Marras, S.A., and Bratu, D.P. (2012). Tiny molecular beacons: LNA/2'-O-methyl RNA chimeric probes for imaging dynamic mRNA processes in living cells. ACS Chem. Biol. 7, 1586-1595.

Chen, X., Zhang, D., Su, N., Bao, B., Xie, X., Zuo, F., Yang, L., Wang, H., Jiang, L., Lin, Q., *et al.* (2019). Visualizing RNA dynamics in live cells with bright and stable fluorescent RNAs. Nat. Biotechnol. 37, 1287-1293.

Deng, S., Stein, R.A., and Higgins, N.P. (2004). Transcription-induced barriers to supercoil diffusion in the Salmonella typhimurium chromosome. Proc. Natl. Acad. Sci. U.S.A. 101, 3398-3403.

Moulin, L., Rahmouni, A.R., and Boccard, F. (2005). Topological insulators inhibit diffusion of transcription-induced positive supercoils in the chromosome of Escherichia coli. Mol. Microbiol. 55, 601-610.

Sobetzko, P. (2016). Transcription-coupled DNA supercoiling dictates the chromosomal arrangement of bacterial genes. Nucleic Acids Res. 44, 1514-1524.

Zuker, M. (2003). Mfold web server for nucleic acid folding and hybridization prediction. Nucleic Acids Res. 31, 3406-3415.

Zuo, F., Jiang, L., Su, N., Zhang, Y., Bao, B., Wang, L., Shi, Y., Yang, H., Huang, X., Li, R., *et al.* (2024). Imaging the dynamics of messenger RNA with a bright and stable green fluorescent RNA. Nat. Chem. Biol.
